# Cervical cancer screening by visual inspection in Côte d'Ivoire, operational and clinical aspects according to HIV status

**DOI:** 10.1186/1471-2458-12-237

**Published:** 2012-03-23

**Authors:** Apollinaire Horo, Antoine Jaquet, Didier K Ekouevi, Badian Toure, Patrick A Coffie, Benjamin Effi, Eugene Messou, Albert Minga, Raoul Moh, Mamourou Kone, François Dabis, Annie J Sasco

**Affiliations:** 1 Service de Gynécologie Obstétrique, Centre Hospitalier Universitaire (CHU) de Yopougon, Abidjan, Côte d'Ivoire; 2 Université Bordeaux, ISPED, Centre INSERM U897-Epidémiologie-Biostatistique, F-33000 Bordeaux, France; 3 INSERM, ISPED, Centre INSERM U897- Epidémiologie-Biostatistique, F-33000 Bordeaux, France; 4 Clinique MTCT + Adultes, ACONDA, Abidjan, Côte d'Ivoire; 5 Programme PAC-CI, CHU de Treichville, Abidjan, Côte d'Ivoire; 6 Service d'Anatomo-Pathologie, CHU de Treichville, Abidjan, Côte d'Ivoire; 7 CePReF, ACONDA, Abidjan, Côte d'Ivoire; 8 Centre Médical de Suivi de Donneurs de Sang/CNTS/PRIMO-CI, Abidjan, Cote d'Ivoire; 9 Département d'infectiologie et de dermatologie, CHU de Treichville, Abidjan, Côte d'Ivoire; 10 Centre de Recherche INSERM U. 897, Institut de Santé Publique, Epidémiologie et Développement (ISPED), Université Bordeaux Segalen, 33076 BORDEAUX Cedex, FRANCE

**Keywords:** Cervical cancer, Screening, Visual inspection, HIV/AIDS, Africa

## Abstract

**Background:**

Cervical cancer screening is not yet standard of care of women attending HIV care clinics in Africa and presents operational challenges that need to be addressed.

**Methods:**

A cervical cancer screening program based on visual inspection methods was conducted in clinics providing antiretroviral treatment (ART) in Abidjan, Côte d'Ivoire. An itinerant team of midwives was in charge of proposing cervical cancer screening to all HIV-positive women enrolled in ART clinics as well as to HIV-negative women who were attending the Abidjan national blood donor clinic. Positively screened women were systematically referred to a colposcopic examination. A phone-based tracking procedure was implemented to reach positively screened women who did not attend the medical consultation. The association between HIV status and cervical cancer screening outcomes was estimated using a multivariate logistic model.

**Results:**

The frequency of positive visual inspection was 9.0% (95% CI 8.0-10.0) in the 2,998 HIV-positive women and 3.9% (95% CI 2.7-5.1) in the 1,047 HIV-negative ones (*p *< 10^-4^). In multivariate analysis, HIV infection was associated with a higher risk of positive visual inspection [OR = 2.28 (95% CI 1.61-3.23)] as well as more extensive lesions involving the endocervical canal [OR = 2.42 (95% CI 1.15-5.08)]. The use of a phone-based tracking procedure enabled a significant reduction of women not attending medical consultation after initial positive screening from 36.5% to 19.8% (*p *< 10^-4^).

**Conclusion:**

The higher frequency of positive visual inspection among HIV-positive women supports the need to extend cervical cancer screening program to all HIV clinics in West Africa. Women loss to follow-up after being positively screened is a major concern in cervical screening programs but yet, partly amenable to a phone tracking procedure.

## Background

Acording to 2008 estimates, invasive cervical cancer (ICC) is the third most common cancer in women worldwide with an estimated 530,232 new cases annually and is responsible for 275,180 deaths. More than 85% of these new ICCs and 88% of these cancer deaths occur in resource-limited settings [1]. In sub-Saharan Africa, a significant association between HIV infection and ICC has been reported, although it has been much less strong compared to reports from resource-replete settings [2]. Since 2002, access to antiretroviral treatment (ART) has been dramatically scaled up with four million HIV-infected patients accessing ART in sub-Saharan Africa at the end of 2009 [3]. As the coverage of ART in low-resource settings continues to improve the prognosis of HIV-infected individuals, it is likely to be translated into years of life saved [4]. Thus, a focus on long-term case management is now needed, especially for women who account for approximately two-thirds of HIV-infected patients receiving ART in sub-Saharan Africa. In high-resource countries, cytology-based cervical screening has curbed the incidence of cervical cancer for decades [5]. Due to human resource and infrastructure shortages, previous experiences in Côte d'Ivoire as well as other sub-Saharan countries highlighted a lack of reliability of cytology-based cervical screening at the population level [6,7]. Cervical screening procedures based on the identification of high-risk human papilloma viruses (HPV) have a high capacity to detect precancerous cervical lesions [8,9]. However, these techniques are currently too work-intensive and expensive to be largely implemented. Low cost, cervical screening procedures based on visual inspection (VI) of the cervix have been proposed and adapted to resource-limited settings for years [10-12]. Although having a lower sensitivity and specificity than HPV tests, they have shown an equal validity compared to cytology in HIV-negative as well as HIV-positive women in resource-limited settings [13,14]. A successful experience with the implementation of such a cervical screening procedure targeting HIV-infected women has been recently reported in Zambia but experiences from other parts of sub-Saharan Africa are limited [15,16]. We therefore sought to compare some of the critical operational and clinical outcomes of a cervical cancer screening based on VI methods among HIV-negative and HIV-positive women in Abidjan, Côte d'Ivoire.

## Materials and methods

### Study population

Our study was a one-shot cervical cancer screening procedure based on visual inspection methods implemented in a sample of HIV care clinics providing ART in Abidjan and involved in "the International epidemiological Database to Evaluate AIDS (IeDEA) in West Africa" http://mereva.net/iedea[17]. Due to logistic and financial constraints, only three of the six adult HIV clinics involved in the IeDEA collaboration were invited to participate. Two nongovernmental organizations, the ACONDA-CePReF (Yopougon) and the ACONDA-MTCT plus (Abobo) together with the national center for blood transfusion (CNTS) (Treichville) were selected in order to cover all the three districts where the IeDEA West Africa sites are already implemented in Abidjan. The CNTS was also selected for its capacity to recruit HIV-negative women. From August 2009 to November 2010, a mobile team composed of three certified midwives and a senior gynaecologist was in charge of proposing cervical cancer screening to all HIV-positive women attending these three clinics for their routine HIV follow-up. The clinics were sequentially involved for a period of six (CePReF, Yopougon), four (MTCT plus, Abobo) and seven (CNTS, Treichville) months, according to the number of women usually attending these services. From May to November 2010, a referent group of HIV-negative women who attended the CNTS clinic for blood donation were also proposed this same cervical cancer screening procedure. Identical exclusion criteria (i.e. history of cervical cancer or total hysterectomy, aged < 25 or > 65 years, pregnancy > 20 weeks) were applied to both HIV-negative and HIV-positive women. Midwives and doctors were not blinded to HIV status.

### Screening procedure

After obtaining written informed consent from the women and collecting participant's history, midwives conducted a gynaecological examination. An unlubricated bivalve speculum was inserted into the vagina by the midwife who examined the cervix using a halogen focus lamp and identified the squamocolumnar junction (SCJ). After cleaning away any excess mucus using a cotton swab, a five percent acetic acid solution was applied to the cervix for visual inspection with acetic acid (VIA). Findings were visible one minute after application. A positive VIA was characterised by a well-defined, dense acetowhite area with regular or irregular margins, close to or abutting the SCJ in the transformation zone or when the whole cervix or a cervical growth turned acetowhite. Following VIA, visual inspection with Lugol's iodine (VILI) was also performed. A positive VILI test was characterised by a dense, thick, bright, mustard-yellow or saffron-yellow iodine non-uptake area seen in the transformation zone, close to or abutting the SCJ or when the entire cervix or a cervical growth turns densely yellow. Results of VIA and VILI were both classified as negative, positive or indicative only of ICC according to the International Agency for Research on Cancer (IARC) training manual and recorded on the study questionnaire following each test [18]. The use of both VIA and VILI for cervical cancer screening relies on previous reports that showed an improvement in diagnostic performance by combining the two methods [19]. Midwives mapped positive lesions with both VIA and VILI on the clinical form and assessed the following items: number of positive lesions, number of quadrants of the cervix involved and if the lesion(s) extend to the endocervical canal. Positively screened women as well as women with inconclusive examination were systematically referred for a colposcopic examination. Women with a negative examination but presenting with cervical inflammatory lesions were systematically referred for repeat VIA and VILI tests three weeks after being prescribed appropriate antibiotics. Prior to the study conduct, midwives were trained to perform and report VIA and VILI during a four-day intensive course. Additional training included discussion and review of pictures of normal and abnormal cervix as well as practice in volunteer subjects. Refresher courses were given during the study period. The colposcopic consultation was carried out in the same clinics where the women were initially screened, on a weekly basis, by a senior gynaecologist. Referred women were assessed with colposcopy and directed biopsies were performed if needed and sent to the Abidjan university hospital pathology unit of Abidjan. Histopathological findings were categorized into five classes: normal or non-neoplasic changes, cervical intraepithelial neoplasia grade 1 (CIN1) including HPV changes, CIN2, CIN3 and ICC as described elsewhere [13]. Women with positive or inconclusive VI were systematically addressed on the same week or a week after to a medical consultation. If not showing up to this first visit, women were contacted by phone calls to schedule another visit. After a maximum of three attempts (one per week), women who did not show-up or who could not be contacted were considered as loss to follow-up (LTFU) and called back once to state their reason for not attending. To enhance participation to the cervical cancer screening in our targeted population and obviate LTFU, women were orally counselled about the risk of cervical cancer and the potential benefits of an early detection. Group counselling was provided by midwives on a daily basis in the waiting rooms of the HIV clinics or the CNTS. Leaflets to sensitize women to cervical cancer and its prevention were also distributed during these sessions. Patients with CIN or cancer were offered appropriate follow-up and treatment according to local recommendations. The entire screening procedure was free of charge as well as the ensuing treatment in case of positive findings. This study was approved by the national ethics committee for HIV research in Côte d'Ivoire.

### Statistical analysis

Analyses were first conducted to compare the women characteristics according to HIV status using Pearson's χ2 or Fisher's exact test, when appropriate, for qualitative variables, and t-test or Wilcoxon test for quantitative variables. A logistic regression model was used for univariate and multivariate analyses of the association between the main outcome of the cervical screening test (i.e. positive visual inspection, inflammatory lesions of the cervix, unsatisfactory examination) and the HIV status. Proportions and odds ratio (OR) estimates were reported with their 95% confidence intervals (95% CIs). All statistical analyses were performed using SAS software, version 9.1 (SAS Institute Inc, Cary, NC, USA).

## Results

### General characteristics

Of the 4,208 women who attended the cervical cancer screening consultations from August 2009 to November 2010, 162 (3.9%) were not eligible for visual inspection tests and excluded. Three per cent of the HIV-positive women were excluded, a significantly lower proportion than the 6.3% figure among the HIV-negative women (*p *< 10^-4^). Among excluded women, 89% of the HIV-negative women were < 25 years compared to 65% in HIV-positive women (*p *< 10^-3^). Six HIV-positive women and two HIV-negative women did not give their consent to participate in the present study. A total of 2,998 HIV-infected women and 1,048 HIV-negative women were effectively enrolled. The baseline characteristics of these women according to their HIV status are summarized in table 1.

**Table 1:** Characteristics of the screened population according to HIV status (n = 4,046) in Abidjan, Côte d'Ivoire.

	HIV+	HIV-	*p*	Total
	N = 2,998	N = 1,048		N = 4,046
Age (years), median [IQR]*	36 [32 42]	34 [29 42]	< 10^-4^	36 [31 42]

Formal education, n (%)			< 10^-4^	
No	757 (25.3)	134 (12.8)		892 (22.0)
Yes	2,241 (74.7)	914 (87.2)		3,154 (78.0)

Monthly income (USD), n (%)			< 10^-4^	
] 40	628 (21.0)	176 (16.8)		804 (19.9)
[40 80[	1,296 (43.2)	305 (29.1)		1,601 (9.6)
[80	794 (26.5)	547 (52.2)		1,341 (33.1)
Unknown	280 (9.3)	20 (1.9)		300 (7.4)

Tobacco use†, n (%)			0.15	
No	2,954 (98.5)	1,039 (99.1)		3,993 (98.7)
Yes	44 (1.5)	9 (0.9)		53 (1.3)

Marital status, n (%)			0.29	
Married or cohabitant	1,345 (44.9)	490 (46.8)		1,835 (45.4)
Single, divorced or widowed	1,653 (55.1)	558 (53.2)		2,211 (54.6)

Number of lifetime sexual partners, n (%)			< 10^-4^	
< 5	1,312 (44.0)	608 (58.4)		1,920 (47.7)
≥ 5	1,669 (56.0)	433 (41.6)		2,102 (52.3)

Age at first sexual intercourse or at			< 10^-4^	
marriage (years), median [IQR]	17 [15 18]	18 [16 19]		17 [15 18]

Parity, n (%)			< 10^-4^	
0	458 (15.3)	336 (32.1)		794 (19.6)
≥ 1	2,539 (84.7)	712 (67.9)		3,251 (80.4)

The IeDEA West Africa collaboration, 2009-2010.

* Inter Quartile Range

† Formal or current consumers of smoked and/or chewed tobacco

The 2,998 HIV-positive women had a median CD4 count of 291 [inter-quartile range (IQR) 156-461] cells/mm^3 ^at enrolment in HIV care and 452 [IQR 301-621] cells/mm^3 ^at last known follow-up visit for HIV infection. The median time between cervical screening and last known CD4 count measure was 4 [IQR 2-6] months. Current ART use was reported in 2,267 (75.6%) HIV-positive women with a median time since ART initiation of 28 months [IQR 12-50].

### Cervical screening outcomes

**Figure 1:**
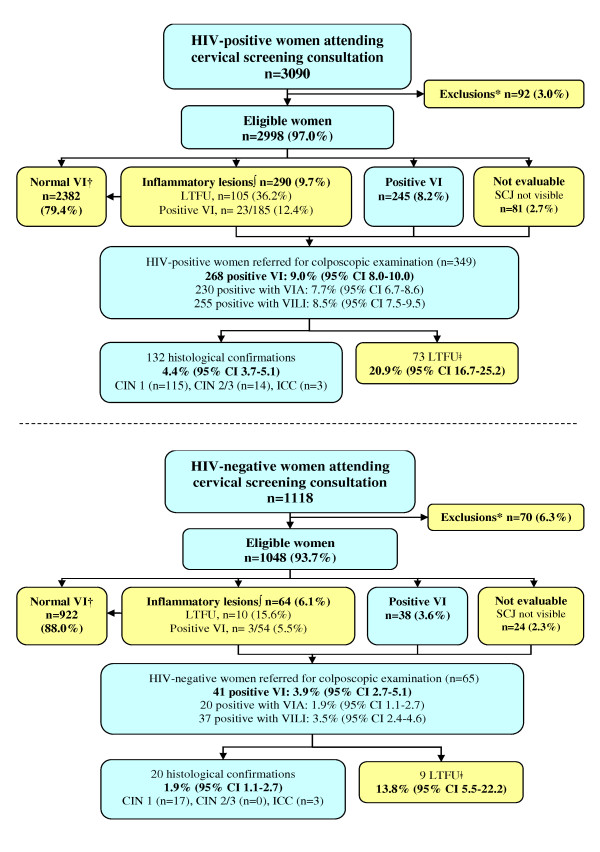
**Patients flow according to HIV status in the IeDEA West Africa cervical screening project in Abidjan, Côte d'Ivoire, 2009-2010**.*Women excluded for at least one of the following criteria: history of cervical cancer or total hysterectomy, aged < 25 or > 65 years, pregnancy > 20 weeks or refusal to participate in the present study †Normal VI Counselled for control at 1 year ∫ Inflammatory lesions referred for other VI tests at 3 weeks ǂ Women addressed to the medical consultation who did not show-up and who could not be contacted after a maximum of three attempts (one per week) were considered as loss to follow-up. Abbreviations: ICC Invasive Cervical Cancer, CIN cervical intraepithelial neoplasia, SCJ squamocolumnar junction, VI visual inspection, VIA visual inspection with acetic acid, VILI visual inspection with Lugol iodine, LTFU loss to follow-up.

The frequency of positive visual inspection in HIV-positive women was 9.0% (95% CI 8.0-10.0), significantly higher than the 3.9% estimate (95% CI 2.7-5.1) found in HIV-negative women (*p *< 10^-4^). Main results of the cervical screening program according to HIV status are summarized in Figure 1.

Of the 414 women referred to colposcopic examination for positive or inconclusive results with VIA and/or VILI, 151 (36.5%) did not attend the initial gynaecologist consultation scheduled during the screening visit. After the application of a systematic phone call tracking procedure to these 151 women, 69 finally attended the colposcopic consultation after being scheduled another appointment. A total of 82 (19.8%) women (73 HIV-positive and 9 HIV-negative) were LTFU. The LTFU rate of women referred for positive or inconclusive visual inspection did not significantly differ in HIV-positive women (20.9%) compared to HIV-negative women (13.8%) (*p *= 0.18). Of the 332 women who effectively attended the gynaecologist consultation and underwent a colposcopic examination, 137 (41.3%) were finally considered as negative. Directed biopsies of the cervix were performed in the remaining 195 women with suspected premalignant or malignant lesions of the cervix. Results were as follow: 132 grade 1 CIN, 14 grade 2 or 3 CIN, six ICC and 43 non-malignant findings. Women with ICC were all refereed to a tertiary hospital and underwent radical hysterectomy except one who died before the intervention. The frequency of precancerous cervical lesions or ICC was 4.4% (95% CI 3.7-5.1) in HIV-positive women and 1.9% (95% CI 1.1-2.7) in HIV-negative women (*p *< 10^-3^). The association between HIV status and the main outcomes of the cervical screening tests is summarized in table 2. HIV-positive women were more than twice as likely to be VI-positive than the negative ones in multivariate analysis (OR = 2.28, 95% CI 1.61-3.23) after adjustment on age, parity and number of lifetime sexual partners. Among the 309 women with positive VI, multiple lesions were found in 27.6% of HIV-positive women and 15.6% of HIV-negative women (*p *= 0.08). Lesions with several quadrants of the cervix involved were reported in 34.1% of HIV-positive women compared to 68.6% in HIV-negative women (*p *< 10^-4^). In HIV-positive women, 50% of the positive lesions at visual inspection extended to the endocervical canal compared to 26.8% in HIV-negative women (*p *< 10^-2^). In multivariate analysis, an association was found between the HIV-positive status and the identification of lesions involving several quadrants of the cervix (adjusted OR = 4.49, 95% CI 2.17-9.28) and the extensions of the lesions to the endocervical canal (adjusted OR = 2.42, 95% CI 1.15-5.08) (Table 2).

**Table 2:** Association between the main outcomes of the cervical screening tests based on visual inspection methods and HIV status in Abidjan, Côte d'Ivoire.

		Univariate			Multivariate*		
	**n/N**	**OR**	**95% CI**	***p***	**OR**	**95% CI**	***p***

Eligible women who underwent a cervical screening with acetic acid and Lugol iodine (n = 4,046)							

Positive VI†				< 10^-4^			< 10^-4^
HIV-negative	41/1,048	1			1		
HIV-positive	268/2,998	2.41	1.72-3.37		2.28	1.61-3.23	

Positive VIA				< 10^-4^			< 10^-4^
HIV-negative	20/1,048	1			1		
HIV-positive	230/2,998	4.27	2.69-6.78		4.02	2.49-6.48	

Positive VILI				< 10^-4^			< 10^-4^
HIV-negative	37/1,048	1			1		
HIV-positive	255/2,998	2.54	1.79-3.61		2.34	1.63-3.36	

Clinical cervicitis				< 10^-3^			< 10^-4^
HIV-negative	64/1,048	1			1		
HIV-positive	290/2,998	1.65	1.24-2.18		1.86	1.38-2.50	

No visualisation of the SCJ				0.47			0.45
HIV-negative	24/1,048	1			1		
HIV-positive	81/2,998	1.18	0.75-1.88		1.2	0.74-1.94	

CIN of any grade‡				10^-3^			< 10^-2^
HIV-negative	20/1,048	1			1		
HIV-positive	132/2,998	2.36	1.47-3.81		2.13	1.31-3.45	

Positively screened women with acetic acid and/or Lugol iodine (n = 309)							

Multiple lesions				0.08			0.10
HIV-negative	6/41	1			1		
HIV-positive	74/268	2.25	0.90-5.51		2.13	0.85-5.35	

Lesions with several				< 10^-4^			< 10^-4^
quadrants of the cervix							
involved							
HIV-negative	14/41	1			1		
HIV-positive	184/268	4.22	2.11-8.47		4.49	2.17-9.28	

Extension to the endocervical canal				< 10^-2^			0.02
HIV-negative	11/41	1			1		
HIV-positive	134/268	2.64	1.27-5.50		2.42	1.15-5.08	

Patients lost to follow-up∫			0.32				0.26
HIV-negative	5/41	1			1		
HIV-positive	50/268	1.65	0.62-4.42		1.89	0.63-5.69	

The IeDEA West Africa collaboration, 2009-2010.

* Adjusted for age group in years ([25-35],[35-45],[45-55] and [55-65]), parity (yes vs. no) and number of lifetime sexual partners (< 5 vs. ≥5)

† Positive visual inspection with VIA and/or VILI

‡ Histological precancerous or cancer lesions confirmed in women who underwent colposcopic examination and directed biopsies

∫ Positively screened women referred for a colposcopic consultation who did not attend the appointment after a telephone-based tracking procedure

Abbreviations: OR Odds ratio, CI Confidence interval, VIA Visual inspection with acetic acid, VILI Visual inspection with Lugol iodine, SCJ Squamocolumnar junction, CIN Cervical intraepithelial neoplasia.

### Loss to follow- up

Among the 82 women LTFU, 64 were reached by telephone and 55 reported the reason for not attending the physician consultation: 39 (70.9%) did not attend for financial reasons (expected cost related to transport, diagnosis and treatment in case of confirmed cervical lesions), 14 (25.5%) for lack of time (being busy or simply forgot) and two women (3.6%) reported not willing to know more about their cervical cancer status.

## Discussion

### Outcomes of cervical screening according to HIV status

A higher rate of precancerous lesions of the cervix or ICC was found in HIV-positive women, consistent with previous reports from sub-Saharan Africa [2]. This finding confirms the need to urgently implement routine cervical cancer screening programs in HIV clinics in West Africa. Regardless of HIV status, the frequency of positive VI and histologically confirmed CIN lesions was relatively low compared to other reports in Africa. In the southern Africa region, a higher frequency of positive VI had been previously reported in HIV-infected women, ranging from 32.6% to 43.7% in South Africa and Zambia, respectively [8,20]. Recent cervical screening program based on VIA conducted in Mali and Tanzania among 14,141 and 10,378 women regardless of HIV status, reported 1,005 (7.1%) and 274 (2.6%) of histologically confirmed CIN, respectively [21,22]. In Botswana, a cervical cancer screening programme based on VIA conducted in 2,175 HIV-positive women reported 331 (15.2%) histologically confirmed CIN [23]. Discrepancies in the frequency of positive VI and precancerous lesions according to VI-based studies might be partly related to the limited reproducibility of these screening methods as well as inclusion criteria such as age that differed according to studies. These regional disparities might also be related to other factors such as cultural differences in sexual life (polygamy, number of sexual partners) that have an influence on sexually transmitted viruses such as oncogenic HPV and finally on the burden of cervical malignancies in sub-Saharan Africa. However, our findings are supported by a recent study conducted in Côte d'Ivoire that found similar frequencies of positive VIA in 2,713 HIV-positive (11%) and 743 HIV-negative women (3%) [24]. Although it remains unclear whether the HIV epidemic has impacted the incidence of ICC in sub-Saharan Africa, it is noteworthy that high ICC incidences are reported in countries with the highest prevalence of HIV [25].

Cervical screening programs based on VIA and/or VILI have previously demonstrated to be an adapted alternative to cytology in both HIV-positive and HIV-negative populations from resource-limited settings [13,14]. To avoid LTFU and tackle human resource shortage for cervical cancer diagnostic and treatment algorithms, a 'see-and-treat' approach proposing an immediate cryotherapy for positively screened cervical lesions with limited extension has been developed and implemented [11,26,27]. Usual criteria for postponing an immediate cryotherapy in a 'see-and-treat' strategy are as follows: lesions occupying > 75% of the transformation zone or more than three quadrants of the cervix, lesions extending into the endocervical canal that cannot be completely visualized and lesions too large to fit the largest available cryotherapy probe [27]. Our study was not initially designed to perform a 'see-and-treat' strategy. Thus, we did not formally address the rate of ineligible women referred for a delayed treatment. However, based on clinical reports of midwives who performed the visual inspection, a high frequency of extensive lesions was observed in our study population, especially in HIV-infected women. This might directly impact on their risk of being in need for a delayed treatment. In Zambia, results from a 'see-and-treat' program using VIA in 8,823 women attending HIV care clinics reported that 2,378 (26.9%) were immediately treated with cryotherapy and 1,477 (16.7%) referred for physician evaluation [20]. In Botswana, a 'see-and-treat' program based on VIA in 2,175 HIV-positive women reported that 253 (11.6%) effectively underwent an immediate cryotherapy and 575 (27.3%) were referred for a delayed medical evaluation [23]. Reports from Ghana and India on 'see-and-treat' programs in the general population found a proportion of ineligible women to an immediate cryotherapy of 2.5% and 25.2%, respectively [26,27]. Thus, the follow-up of ineligible women for an immediate treatment remains a major issue, especially in HIV-positive populations.

### Loss to follow-up

The proportion of positively screened women who did not attend the medical consultation prior to the application of a tracking system is consistent with a previous report from Zambia where 40.8% of positively screened women ineligible for an immediate cryotherapy and referred to a physician examination did not show up [20]. The adjunction of a simple tracking procedure based on telephone calls inviting to the mobile medical consultation for colposcopic examination allowed a major reduction in the proportion of women LTFU. However, a significant rate of LTFU remained. A particular effort must be done to sensitise women to the importance of cervical malignancies and the advantages of an early identification of precancerous lesions of the cervix. Finally, the implementation of a 'see-and-treat' approach should be incorporated into cervical screening in our West African network considering the experience reported in Zambia among others. However, even if a single visit approach is used, an important proportion of HIV-positive women presenting with more extensive lesions will not be eligible for this 'see-and-treat' approach. Additional research is needed in order to more efficiently reduce the attrition proportion in cervical cancer screening programs. Studies exploring reasons for being LTFU as well as alternative interventions to minimize it need to be developed.

### Limitations

Because only women who were VI positive underwent histological sampling, the performance of VI could not be assessed. Accuracy studies on VI showed contrasting results. While a recent meta-analysis mostly based on observational studies reports a 80% sensitivity (range, 79%-82%) and a 92% specificity (range, 91%-92%) of VIA in detecting high-grade precancerous lesions [28], several randomised trials have shown poorer performance of VIA (sensitivity of 55%) compared to HPV testing (sensitivity 90%) in both HIV-negative and HIV-positive women [9,29]. As cytology or HPV testing are currently not available, we believe that VI is the most appropriate approach that can at least promote the awareness and initiate the infrastructure for building cervical cancer screening in HIV clinics in Côte d'Ivoire. However, the true performance of VI in this particular context remains to be evaluated. The results reported from this sample of HIV care clinics might not be directly extrapolated to other clinics in West Africa, especially those not located within the proximity of a referral hospital. Nevertheless, the participation of clinics of various sizes and from different health care levels enabled us to extend our findings to at least the HIV care system of Côte d'Ivoire. VI of the cervix remains highly related to the operator skills and subject to misclassification bias. Despite an adapted initial training of midwives associated with refresher courses during the study period, a high and sustained proportion of positively screened women were considered negative after colposcopic examination and did not undergo directed biopsy. The rate of false positivity according to colposcopic results was higher compared to prior accuracy studies conducted by IARC in Africa and India were 2,887 (33%) of the 8,848 women with positive VIA were recused for a directed biopsy [12]. These differences probably reflect the wide range in accuracy parameters (Se, Sp, PPV and NPV) of VI related to the subjective, provider-dependent nature of the test and the underlying frequency of disease. However, when considering a 'see-and-treat' approach based on VI, a significant part of positively screened women might be over-treated with cryotherapy. Although the short-term safety of cryotherapy performed immediately after VI has been relatively well addressed, its long-term safety, especially in HIV-infected women remains to be explored [30]. Alternative screening strategies including more accurate and reproducible tests such as the rapid HPV test will ultimately be needed to enhance the performance of screening for the identification of precancerous lesions.

## Conclusion

We confirm that HIV-infected women are at higher risk of presenting a positive cervical screening test as well as histological precancerous lesions compared to HIV-negative women, highlighting the need to extend cervical cancer screening to all HIV care clinics in West Africa. Specific issues related to visual inspection outcomes according to HIV status were identified such as more extensive cervical lesions in HIV-positive women, leading to a potentially lower number of women eligible for a 'see-and-treat' approach. Regardless of HIV status, LTFU was a main barrier limiting the sustainability of this cervical cancer screening approach. However, the use of a simple telephone recall enabled a significant reduction in LTFU. Reasons reported by women who did not attend colposcopic consultation suggested that interventions to sensitise women to the benefits of an early detection and treatment of precancerous cervical lesions are essential to prevent LTFU. Additional studies exploring the determinants associated with LTFU as well as alternative interventions that could significantly impact on LTFU need to be developed.

## Competing interests

The authors declare that they have no competing interests.

## Authors' contributions

AH, AJ, DKE, FD and AJS designed the study. AH and BT conducted the clinical work and cervical screening training. AJ, DKE and AH supervised the study. BE supervised the pathological examinations. Statistical analysis was done by AJ and interpretation of data was done by AH and AJ The manuscript was drafted by AJ and critical revision of the manuscript for important intellectual content was provided by AH, AJS, DKE, PAC, RM and FD All authors read and commented on the original manuscript and all agreed on the version finalised by AJ for submission.

## Funding

This work was funded by the following institutes: the National Cancer Institute (NCI), the Eunice Kennedy Shriver National Institute of Child Health & Human Development (NICHD), the National Institute of Allergy and Infectious Diseases (NIAID) (grant n° 5U01AI069919) and the ANRS: « Agence Nationale de Recherches Sur le SIDA et les hépatites virales » (ANRS 12136 Temprano)

## Pre-publication history

The pre-publication history for this paper can be accessed here:

http://www.biomedcentral.com/1471-2458/12/237/prepub
